# The Structural Biology of Bcl-x_L_

**DOI:** 10.3390/ijms20092234

**Published:** 2019-05-07

**Authors:** Erinna F. Lee, W. Douglas Fairlie

**Affiliations:** 1 Department of Biochemistry and Genetics, La Trobe Institute for Molecular Science, La Trobe University, Bundoora, Victoria 3086, Australia; 2 Olivia Newton-John Cancer Research Institute, Heidelberg, Victoria 3084, Australia; 3 School of Cancer Medicine, La Trobe University, Melbourne, Victoria 3084, Australia

**Keywords:** apoptosis, Bcl-2, Bcl-x_L_, BH3-only, BH3 domain, BH3-mimetic, pro-survival, structural biology

## Abstract

Interactions between the pro-survival and pro-apoptotic members of the Bcl-2 family of proteins dictate whether a cell lives or dies. Much of our knowledge of the molecular details of these interactions has come from biochemical and structural studies on the pro-survival protein Bcl-x_L_. The first high-resolution structure of any Bcl-2 family member was of Bcl-x_L_, which revealed the conserved topology amongst all family members. Subsequent structures of Bcl-x_L_ complexes with pro-apoptotic ligands demonstrated the general features of all pro-survival:pro-apoptotic complexes. Structural studies involving Bcl-x_L_ were also the basis for the discovery of the first small-molecule pro-survival protein inhibitors, leading ultimately to the development of a new class of drugs now successfully used for cancer treatment in the clinic. This article will review our current knowledge of the structural biology of Bcl-x_L_ and how this has impacted our understanding of the molecular details of the intrinsic apoptotic pathway.

## 1. Introduction

Correct regulation of the intrinsic apoptotic pathway by the Bcl-2 family of proteins is essential for the normal development and physiology of all multi-cellular organisms. Central to the pathway are the Bcl-2 pro-survival proteins (Bcl-x_L_, Bcl-2, Bcl-w, Bfl-1, Mcl-1) that inhibit apoptosis by directly engaging their pro-apoptotic counterparts, the effector proteins Bax, Bak (and possibly Bok), or the upstream initiators of the pathway, the BH3-only proteins (Bim, Puma, Bid, Noxa, Bad, Bmf, Hrk, Bik) [1]. The prototypical family member is Bcl-2 after which the family was named. Its discovery, based on its abnormal expression in B-cell lymphomas [2,3,4], subsequently led to landmark experiments demonstrating the pro-survival function of the Bcl-2 protein, and the first dissociation of cell survival from cell proliferation pathways [5]. However, extensive structural studies of its close relative, Bcl-x_L_, have perhaps provided the greatest insights into the molecular mechanisms by which the intrinsic apoptosis pathway is regulated. Indeed, the first three-dimensional structural data for any family member came from studies on Bcl-x_L_ (refer to Table 1 for Protein Data Bank (PDB) and related information for all structures described throughout the text) [5]. Subsequent studies showing how Bcl-x_L_ binds to its natural ligands, the BH3 domains of pro-apoptotic family members, enabled us to visualise the conserved mechanism by which all of the pro-survival proteins can engage both sub-classes of pro-apoptotic molecules. These structures in turn paved the way for the development of the first drugs targeting Bcl-2 pro-survival proteins that are now showing success in the clinic [6,7,8]. In this article we will review the now extensive literature on Bcl-x_L_ structural biology, and how this has informed both our understanding of the molecular events that govern apoptosis regulation, and been applied in drug development.

## 2. Bcl-x_L_ Primary Structure

The *BCL-X* gene was first cloned in 1993 based on its similarity to *BCL-2* due to the capacity of a murine *bcl-2* cDNA to hybridise to a related chicken gene [52]. This sequence was then used to isolate the human *BCL-X* ortholog. In the same year, the genes for *MCL1*, *A1* and *BAX* were also cloned [53,54,55], and by 1996, all of the core human pro-survival proteins and effector proteins had been discovered [56,57,58,59] (though additional Bcl-2 homologues such as Bcl2L10/Bcl-B were subsequently identified). It soon became apparent that all of these proteins contain four conserved regions of sequence homology that are known as the Bcl-2 Homology (BH) domains 1 to 4, as well as a hydrophobic C-terminal region predicted to act as a transmembrane anchor [52,53,54,55,56,57,58,59,60,61]. When the *BCL-X* gene was identified, another shorter isoform (termed Bcl-x_S_) was also isolated that encodes a protein lacking the BH1 and BH2 domains, but possessing both the BH3 domain and hydrophobic tail [52]. The third sub-class of the family, the pro-apoptotic BH3-only proteins, only possesses a BH3 domain, though some also have transmembrane anchors at their C-terminus. 

Among the multi-BH domain proteins, Bcl-x_L_ and Bcl-2 have the longest sequences in the region that spans the BH domains (although Mcl-1 has a very long N-terminal extension, making it the longest sequence overall). This is due to both Bcl-x_L_ and Bcl-2 possessing a longer sequence connecting the N-terminal region containing the BH4 domain and the BH3 domain, compared to the other family members. Bcl-x_L_ shares the highest sequence identity with Bcl-w (51%) and Bcl-2 (45%), with all the other multi-BH domain proteins being just 18–25% identical. Shortly after the cloning of all of these genes, the first three dimensional structure of any Bcl-2 family member was determined. This structure provided the first insights into the roles of each of the conserved BH domains, and more importantly, provided the first clues on how Bcl-2 family proteins interact with each other to regulate apoptosis.

## 3. *Apo* Bcl-x_L_ Structures

The first three-dimensional structure of a Bcl-2 family protein was that of human Bcl-x_L_ [5]. This was determined by both nuclear magnetic resonance (NMR) and X-ray crystallography, with good agreement between the structures. Subsequently, X-ray crystal structures of mouse and rat Bcl-x_L_ were also reported [9,11]. In each case (and in most, though not all, structures of Bcl-2 proteins solved to date [see below for some exceptions]), the constructs used lacked the C-terminal transmembrane domain, primarily to facilitate solubility of the protein at the high concentration required for structural studies.

**Figure 1:**
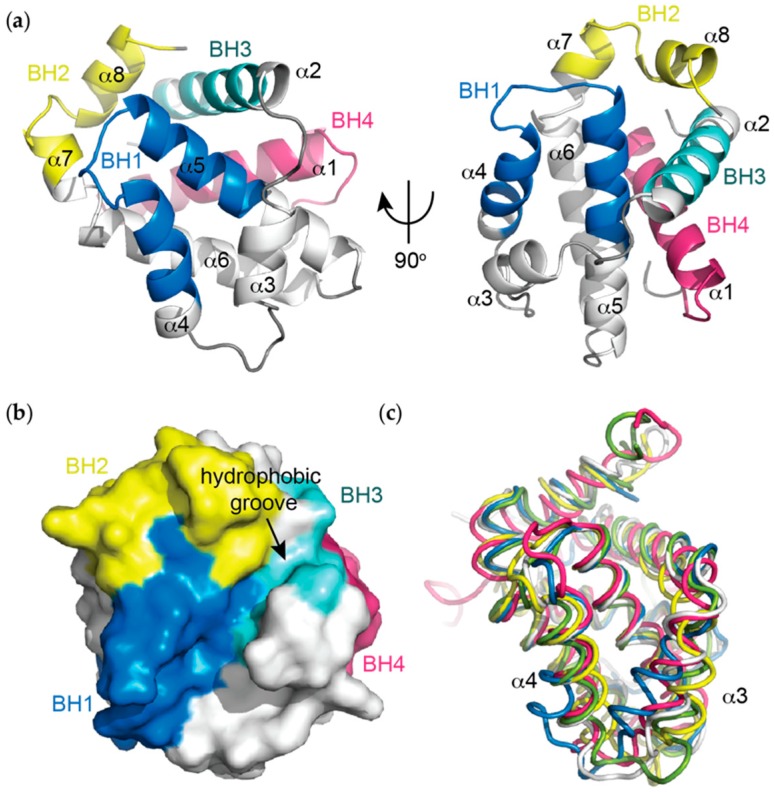
(**a**) Helical representation of *apo* BCL-X_L_ (PDB ID: 1MAZ). The BCL-2 homology (BH) domains (coloured), make significant contributions to defining the tertiary structure of BCL-2 pro-survival proteins. The α5 and α6 helices form a central hairpin surrounded on either side by the other helices; (**b**) Surface representation of *apo* BCL-X_L_ demonstrating the canonical hydrophobic binding groove created mainly by helices α3 and α4 with α5 forming the base, which is critical for mediating interactions with the pro-apoptotic proteins of the BCL-2 family; (**c**) Overlay of *apo* structures of the pro-survival members of the BCL-2 family demonstrating idiosyncrasies in the orientations of the α3 and α4 helices that line the hydrophobic groove. In particular, notable differences in the orientation of α3 and α4 are observed. Bcl-x_L_ (PDB ID: 1MAZ, blue); Bcl-2 (PDB ID: 1GJH, white); Bcl-w (PDB ID: 1O0L, green); Mcl-1 (PDB ID: 1WSX, pink); Bfl-1 (PDB ID: 5WHI, yellow).

The Bcl-x_L_ structure consists of eight alpha helical regions (α1-α8) (Figure 1a) (note: the original structure paper delineated seven helices [5], though the short helical segment at the C-terminal end of α6 is generally referred to as a separate helix in subsequent Bcl-2 family structural papers). Helices α5 and α6 form a central hairpin arrangement that is flanked by α3 and α4 on one side and α1, α2 and α8 on the other. This arrangement of the central α5-α6 helices, together with the surrounding α1, α3 and α4 helices is reminiscent of membrane insertion domains of the pore forming toxins including diphtheria toxin [5,62]. In the X-ray crystal structure there is no electron density for the extended region connecting α1 to α2 consistent with the NMR data for that region, suggesting that it adopts a flexible random coil structure [5]. 

In the context of the three-dimensional structure of Bcl-x_L_, the BH domains make essential contributions to its tertiary structure (Figure 1a,b). The BH1 and BH2 domains encompass turn regions linking two helices, α4 to α5 (in the case of BH1) and α7 to α8 (in the case of BH2). The BH3 domain is located entirely on α2 whilst the BH4 domain is located on α1 and makes a number of stabilising hydrophobic contacts with α2, α5, and α6. 

Structures of all the pro-survival Bcl-2 family members in the *apo* form have now been determined and they all possess the same overall topology despite relatively low sequence identity in many cases [63,64,65,66] (Figure 1c). Remarkably, the pro-apoptotic proteins Bax, Bak and Bok also adopt similar structures [67,68] despite their diametrically opposing functions. Most BH3-only proteins are intrinsically disordered [69], though Bid also has a similar structural topology to the multi-BH domain Bcl-2 homologues [70].

A major structural feature noted on the first Bcl-x_L_ structure is a large hydrophobic groove involving the BH1-BH3 domains (Figure 1b). This is formed predominantly between helices α3 and α4, with α5 lining the base of the groove, though residues from the BH2 (α8) and BH3 (α2) domains also contribute. The hydrophobic groove represents the region of greatest difference between the pro-survival proteins (Figure 1c). In Bcl-x_L_, α3 and α4 are almost parallel and are relatively tightly packed (probably due to a hydrogen bond between Gln-111 on α3 and Glu-129 on α4) compared to in other structures (e.g., Mcl-1 or Bfl-1) where the helices adopt more of a V-shape, resulting in a more open groove (Figure 1c). Early mutagenesis studies suggested this cleft could be the site of interaction with pro-apoptotic proteins [61], which was subsequently confirmed when the first structures of Bcl-x_L_ complexes with pro-apoptotic ligands were determined.

## 4. Bcl-x_L_ Heterodimer Structures with BH3 Domains

### 4.1. Canonical BH3 Domains

**Figure 2:**
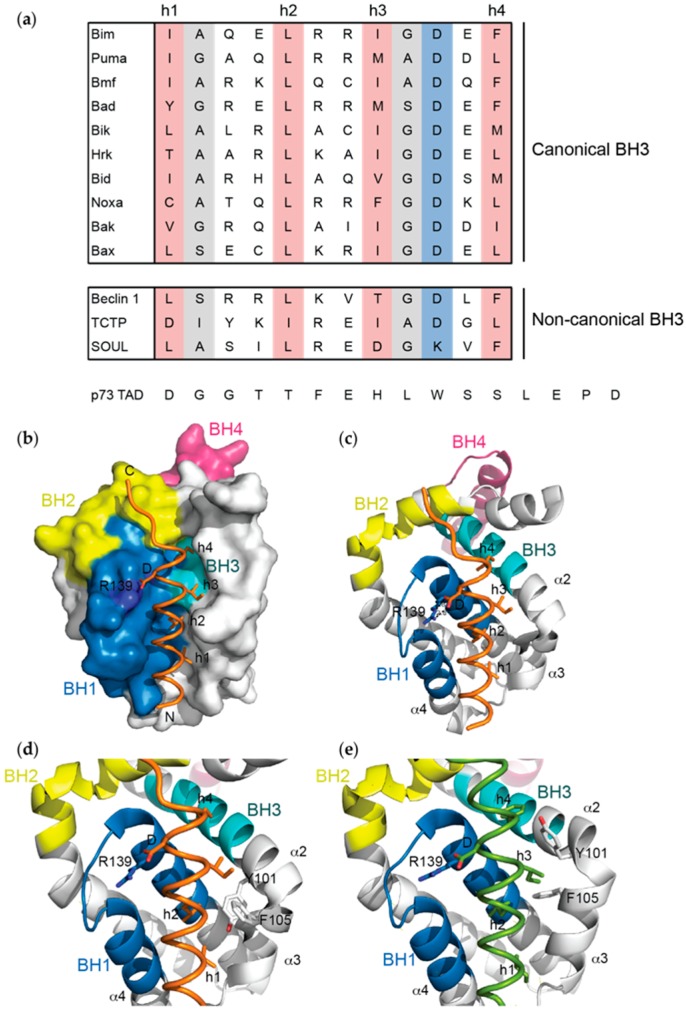
(**a**) Sequence alignment of BH3 domains. Canonical BH3 domains that bind with high affinity to pro-survival proteins are defined by the presence of four conserved hydrophobic residues (h1 to h4, pink), an invariable aspartic acid (blue) and amino acid residues with smaller side-chains in positions highlighted in grey. In contrast, the affinities of non-canonical BH3 domains are weaker due to non-conserved amino acid substitutions at these key positions. The p73 TAD sequence that binds Bcl-x_L_ with weak affinity has no sequence homology to BH3 domains; (**b**,**c**) The structure of the Bak BH3 domain (orange) in complex with Bcl-x_L_ defined the conserved mechanism by which pro-apoptotic proteins bind their pro-survival targets (PDB ID: 5FMK)—the four conserved hydrophobic residues (h1–h4) project into pockets along the hydrophobic groove, the invariant aspartic acid forms an electrostatic interaction with a conserved arginine in the BH1 domain of Bcl-x_L_, and the BH3 domain forms an amphipathic helix that binds into the groove; (**d**,**e**) The orientation of Tyr-101 and Phe-105 of Bcl-x_L_ changes relative to the hydrophobic groove depending on the BH3 domain that engages it (e.g., (**d**) Bak BH3 (orange):Bcl-x_L_, PDB ID: 5FMK; (**e**) Bim BH3 (green):Bcl-x_L_, PDB ID: 3FDL). Notably, these residues lie in the α2-α3 corner of Bcl-x_L_ that is associated with significant conformational change when BH3 domains bind.

Early structure-function studies on both the multi-BH domain proteins Bak and Bax and BH3-only proteins Bik and Bad identified the BH3 domain to be the principle site of interaction with Bcl-x_L_ and/or Bcl-2 [60,71,72]. This was subsequently shown to be true for all pro-survival:pro-apoptotic protein interactions. The BH3 domain is a short sequence motif primarily defined by four hydrophobic resides at positions referred to as h1 to h4 [73] (h2 is always a leucine in pro-apoptotic BH3 domains), a conserved aspartic acid residue, and typically residues with smaller side-chains (glycine and alanine) at positions h1+1 and h3+1 (Figure 2a).

Using NMR, the complex of Bcl-x_L_ with a 16-residue peptide encompassing the Bak BH3 domain was the first three dimensional structure of any Bcl-2 protein complex to be determined [22]. The BH3 peptide bound as an amphipathic α-helix within the hydrophobic groove (Figure 2b,c), as expected based on early structure-function studies, with the N-terminus of the peptide oriented towards the α3-α4 apex. The four conserved hydrophobic residues are aligned along one face of the helix and project into the groove where they are accommodated within small hydrophobic pockets (Figure 2b,c). For example, the conserved leucine residue (h2) engages a pocket formed by Tyr-101, Leu-108, Val-126 and Phe-146 on Bcl-x_L_. The highly conserved aspartic acid residue (Asp-83 on Bak) makes an electrostatic interaction with Arg-139 on Bcl-x_L_, a residue within the BH1 domain that is also conserved on all pro-survival proteins. Mutation of any of these residues on Bak reduced affinity for Bcl-x_L_, whilst other contacts between the Bak peptide and Bcl-x_L_ made smaller contributions to the overall affinity of the interaction [22]. Nevertheless, a subsequent X-ray crystal structure of a longer Bak BH3 peptide bound to Bcl-x_L_, together with that of a Bak BH3 mutant (Q75L) provided a rationale for the slightly weaker affinity conferred by this mutation (i.e., loss of a hydrogen bond) that also has profound physiological effects when introduced into mice [21].

Over the years there have been multiple NMR and X-ray crystal structures reported for complexes between Bcl-x_L_ and natural pro-apoptotic BH3 domain peptide ligands (Table 1) including Bim, Puma, Bid, Bad and Bax (note there are no structures of a full-length BH3-only protein, or Bax/Bak protein, in complex with any pro-survival protein to date) [10,11,17,18,19,23]. In each case the overall interaction is similar to that seen with the Bak BH3 domain, with the four conserved hydrophobic residues projecting into the groove and an electrostatic interaction between the conserved aspartate and Arg-139 on Bcl-x_L_. However, most structures also display unique features consistent with differences between the BH3 sequences. For example, in Bax there is a fifth hydrophobic residue (Met-78) that is required for high affinity binding [23], whilst in Bid BH3, a unique histidine residue at the h2-1 position makes a π-stacking interaction with Phe-105 on Bcl-x_L_ not seen in other structures [49].

**Figure 3:**
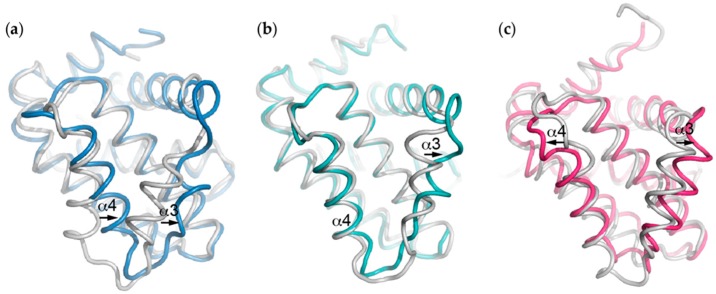
Movement of the α3 and α4 helices that form the “walls” defining the canonical ligand-binding groove to accommodate BH3 ligand binding differs between the different pro-survival proteins. Comparison of the structures of the *apo* pro-survival proteins (white) with just the pro-survival target from the BH3-bound form (i.e., with ligand removed) (**a**) Bak BH3:Bcl-x_L_ (PDB ID: 1BXL, blue), (**b**) Bax BH3:Bcl-2 (PDB ID: 2XA0, aqua) and (**c**) Bim BH3:Mcl-1 (PDB ID: 2NL9, pink) complexes exemplify this.

Another notable difference is the disposition of Phe-105 which varies somewhat between structures. In some complexes (e.g., with Bak BH3) it is orientated out of the groove, whereas in other complexes it lines the h2 pocket, though in different orientations (Figure 2d,e). The orientation of nearby Tyr-101 also changes relative to Phe-105; projecting in opposite directions to each other relative to the groove (Figure 2d,e). Both Tyr-101 and Phe-105 are located at the α2-α3 corner, the region associated with the major conformational change that occurs in Bcl-x_L_ upon BH3 peptide binding. Interactions with BH3 peptides induces an opening of the hydrophobic groove where α3 shifts away from the peptide and in most cases becomes less helical and disordered, though to different degrees depending on the peptide (Figure 3a). The α4 helix also moves, though towards the peptide, and overall the groove becomes more open and V-shaped. This is somewhat different to in other Bcl-2 family protein:BH3 peptide complexes. For example, in the Bcl-2:Bax BH3 complex [74], it is mostly α3 that moves to accommodate the peptide (Figure 3b) whereas in the Mcl-1:Bim BH3 complex, the α3 and α4 helices at the top of the open V-shaped groove in the *apo* protein move slightly away from the peptide upon binding [75] (Figure 3c). Perhaps the most dramatic effects upon BH3 peptide binding is observed when the Puma BH3 peptide binds Bcl-x_L_. Both α2 and α3 become highly disordered, particularly around the α2-α3 corner, and this is proposed to have important functional consequences (discussed in detail below in section on interactions with p53) [10].

### 4.2. Non-Canonical BH3 Domains

**Figure 4:**
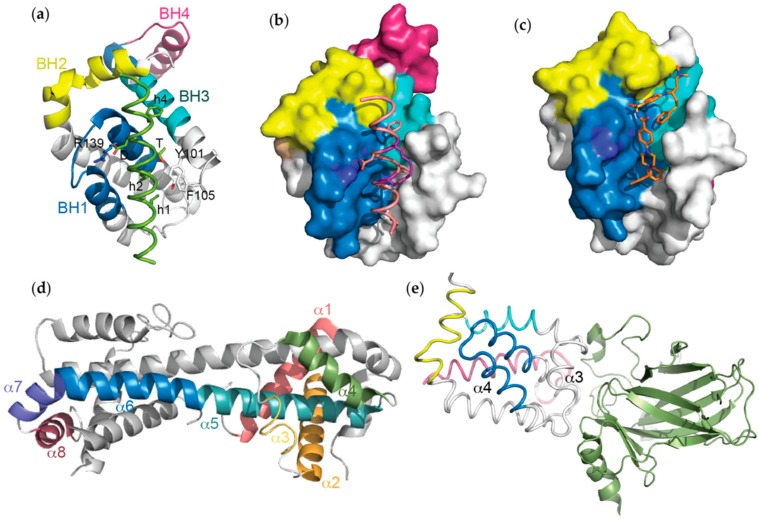
The mode of binding between representative (**a**) non-canonical BH3 domains (Beclin 1 BH3 (green):Bcl-x_L_, PDB ID: 2P1L), (**b**) unnatural peptides (BimSAHB (orange/hydrocarbon staple in purple):Bcl-x_L_, PDB ID: 2YQ6) and (**c**) small molecule BH3-mimetics (ABT-737 (orange):Bcl-x_L_, PDB ID: 2YXJ) to Bcl-x_L_ is highly conserved, with all ligands engaging some or all of the hydrophobic pockets that line the conserved groove of the pro-survival protein. These pockets are normally targeted by the conserved hydrophobic residues (h1 to h4), which define BH3 domains, to confer high affinity binding. Significant conformational changes in Bcl-x_L_ also occur following exposure to certain stimuli such as (**d**) heat (PDB ID: 2B48) where the helices undergo major rearrangements or (**e**) following binding of p53 to an acidic patch adjacent to the Bcl-x_L_ hydrophobic groove, resulting in movements in the helices surrounding it (PDB ID: 2MEJ).

Beyond complexes with pro-apoptotic BH3 domains, structures of Bcl-x_L_ bound to other less well-conserved BH3-like sequences have also been reported. Probably the interaction that is best supported by biological data is with a key regulator of autophagy, Beclin 1 [76,77]. Beclin 1 possesses a BH3 domain which has been captured bound to Bcl-x_L_ in NMR and X-ray crystal structures [26,27]. All demonstrate the same binding mode as the pro-apoptotic BH3 domains, and together with mutagenesis data, suggested that an unusual polar residue (threonine) in place of the third conserved hydrophobic position (h3) of Beclin 1, accounts for its somewhat weaker affinity (low µM *versus* nM) compared to sequences such as Bim and Bad. These structures showed a similar orientation of Phe-105 and Tyr-101 as in the complex with Bak BH3, likely due to both BH3 domains having a hydrophobic residue at the h3-1 position rather than a hydrophilic residue seen in most other BH3 domains (Figure 2a and Figure 4a). In the X-ray crystal structure, Bcl-x_L_ was observed as a dimer formed by swapping α1 helices between protomers [27]. This dimer was not observed in the NMR structure of the same complex [26]. This difference is due to the use of a Bcl-x_L_ construct in which the α1-α2 loop was significantly truncated to aid in crystallisation for the X-ray structure. Due to its apparently greater propensity to crystallise with other BH3 peptides, this construct has now been used to determine multiple structures with different BH3 domains. More recently X-ray crystal structures informed the mechanism by which the kinase Mst1 regulates autophagy through phosphorylation of Thr-108 in the Beclin 1 BH3 domain [32].

The shorter isoform of Bcl-x_L_, Bcl-x_S_, as well as a caspase-cleaved form of Bcl-x_L_ have been reported to possess pro-apoptotic activity due to their ability to bind Bcl-x_L_ *via* their BH3 domains [78,79]. Biochemical and mutagenesis data confirmed this interaction of Bcl-x_L_ with its own BH3 domain, though showed it was relatively weak (0.6 µM affinity) due to the presence of a long side-chain (lysine) at the h1+1 position that is typically a smaller residue in pro-apoptotic BH3 domains [28] (Figure 2a). A structure of this complex again showed the conserved binding mode, though it also explained the weak affinity as the large lysine side-chain at h1+1 projects out of the binding groove and forces the N-terminus of the BH3 domain to adopt a conformation that prevents it (especially the h1 residue) from being buried, as in all other BH3 structures.

A highly divergent BH3 domain (no h1, Ile instead of Leu at h2, Figure 2a) from Translationally Controlled Tumour Protein (TCTP) has also been crystallised in complex with Bcl-x_L_ [29]. This complex shows most of the usual features of BH3 domain complexes (burial of h2, h3, h4 in the groove) and an electrostatic interaction with Arg-139. However, there were essentially no contacts preceding h2, accounting for the very low affinity (10 µM) of this interaction. Yet another protein, SOUL that can induce the mitochondrial permeability transition, also has a non-canonical BH3 domain (aspartate at h3 and lysine in place of the conserved aspartate, Figure 2a) with very weak affinity (~40 µM) for Bcl-x_L_. A structure of this peptide bound to Bcl-x_L_ shows the typical binding mode of other BH3 domains though lacks both the h3 hydrophobic contact and the electrostatic interaction with Arg-139, consistent with the low affinity of the interaction [30]. Most unusual of these non-canonical structures is the complex between Bcl-x_L_ and a 16-residue peptide from the transactivation domain of the transcription factor p73 (p73 TAD) that can induce transcription-independent apoptosis [31]. The only resemblance of this peptide to BH3 domains is its amphipathic nature (Figure 2a). The structure shows it engages the hydrophobic groove, and although it makes contact with residues on the BH1, BH2 and BH3 domains, these interactions are only at the very “top” portion of the groove where the C-terminus of BH3 peptides normally bind, and results in a displacement of α8. The peptide is also orientated in the opposite direction to that of canonical BH3 domains. Whilst these structures are interesting for showing the flexibility of Bcl-x_L_ for binding to diverse ligands, the physiological relevance of these complexes still requires further investigation.

### 4.3. Non-Natural BH3 Peptide Complexes

The high affinity and specificity of some peptides, including those of BH3 domains, suggests that they could have potential for therapeutic applications. However, their inability to cross-cell membranes to engage intracellular targets (such as Bcl-2 family proteins) and their susceptibility to proteolysis limits such applications. Nevertheless, several approaches have been used to improve these properties of BH3 peptides to make them more “drug-like”. In this context, Bcl-x_L_ has been a target of some of these modified sequences, with structural studies being useful for their design and biophysical characterisation.

One approach that has been used for multiple Bcl-2 protein targets including both pro-survival and pro-apoptotic family members is the installation of “hydrocarbon staples” on the non-binding surface of the BH3 domain α-helix [65,80,81,82]. Such staples are purported to have multiple benefits including increasing resistance to proteolysis, increasing target affinity due to favourable energetic parameters associated with pre-organising the α-helix, as well as enabling the peptide to cross cell membranes. Interestingly, a study focussing mostly on a stapled Bim BH3 peptide and its interaction with Bcl-x_L_ (Figure 4b) showed that introduction of the staple unexpectedly reduced affinity for Bcl-x_L_ compared to the native Bim sequence [37]. Whilst there was some controversy over the nature of the sequences used in this study compared to prior reports [37,83], structural studies showed that although the staple did not make any unfavourable contacts with the target, it prevented formation of important intra-peptide non-covalent interactions [37]. A subsequent structural and biophysical study also showed stapling had adverse effects on the Bim BH3:Bcl-x_L_ interaction though they proposed a more complex model based on the binding energetics to account for this weakened affinity [36].

A second approach to increasing resistance to proteolysis is to incorporate β-amino acids into a peptide sequence. These amino acid residues possess one extra carbon atom in their backbone compared to α-amino acids and are not readily recognised by proteases, rendering peptides in which they are incorporated more resistant to enzymatic degradation. The BH3 domain has been extensively used as a model system to understand the optimal arrangement of such residues for maximal binding affinity [17,33,34,35,84,85]. In general, the most efficient approach to designing such peptides is to incorporate one β-residue per turn of the helix such that these residues align on the solvent exposed surface upon target binding, though β-residues within the binding interface can also be tolerated in some cases [33,84,86]. Structural studies of α/β-BH3 (e.g., Bim and Puma) peptide complexes with Bcl-x_L_ (and other pro-survival proteins) have shown remarkable mimicry of the canonical interactions seen with native BH3 domain peptides despite the addition of approximately one extra carbon atom per helical turn [17,33,34,35]. Detailed analyses of these structures have shown that the extra backbone carbon atoms are accommodated due to a combination of small changes to the peptide structure including increased helix radius and reduced phase yield and rise per α-residue in the α/β-sequences [34]. Interestingly incorporation of β-amino acids can alter the selectivity of BH3 domain peptides compared to all-α peptides despite having identical side-chain chemistry [33,34,35,84]. Structures demonstrated that affinity variations for different targets arise due to changes in the orientation of solvent exposed residues that can sometimes lead to steric clashes with the side-chains of adjacent residues on the target. Nevertheless, such structures were also critical for the successful design of new sequences where native peptide specificity is recapitulated [35]. In one case, this involved design of an α/β-peptide that also incorporated a D-amino acid as well as a homonorleucine in place of the h2 leucine residue to more effectively fill the h2 pocket.

Other BH3 peptides have also been designed with non-natural amino acids to promote different functionalities. For example, Bim BH3 peptides with a novel extended side-chain at the h3 position were generated to create a Bak inhibitor [38]. A structure of Bcl-x_L_ bound to one of these peptides was important to facilitate design of further sequences to reduce Bcl-x_L_ binding and thereby enhance their anti-apoptotic effect. Another peptide based on Bak BH3 was modified to incorporate a photoactivatable “switch” that increases affinity for Bcl-x_L_ by stabilising the helix [14]. A structure of this peptide in complex with Bcl-x_L_ showed that the peptide-stabilising crosslinker induces a slight shift in the register of the peptide helix along the groove relative to wild-type Bak BH3, with concomitant remodelling of the groove to retain favourable interactions with the peptide.

### 4.4. Membrane-Bound Bcl-x_L_

All structures of Bcl-x_L_ described above lack the C-terminal transmembrane domain due to difficulties producing the full-length protein in a soluble form at concentrations required for structural studies. This technical issue has, therefore, precluded structural studies on the membrane-bound form of Bcl-x_L._ However, two recent structural studies have begun to address the important question of how membranes affect the structure and function of pro-survival proteins given this is their natural cellular milieu [51,87,88]. Both studies use NMR-based techniques and employ nanodiscs to serve as a membrane surrogate. In the first study, Bcl-x_L_ with its transmembrane domain (though lacking part of its unstructured loop) was purified directly from bacteria in a soluble form [87,88]. NMR studies in aqueous solution showed that the transmembrane region interacts with the hydrophobic binding groove. A similar interaction of the C-terminus of Bcl-w with its hydrophobic groove has previously been observed though, in Bcl-x_L_, this appears to be more dynamic. The binding affinity of the full-length Bcl-x_L_ to a BH3 peptide from Bid was reduced compared to just the soluble domain without the transmembrane helix, indicative of the need to displace the transmembrane helix, and this was confirmed by NMR chemical shift data. Surprisingly, NMR studies on the full-length Bcl-x_L_ integrated into nanodisc lipid bilayers showed that the overall structure of Bcl-x_L_ is very similar to that of the soluble form without its tail region, contrary to earlier NMR studies using detergent micelles as membrane surrogates [89], and it retains high affinity binding to a Bid BH3 peptide. The second study used a SortaseA-ligation approach whereby the Bcl-x_L_ soluble domain was ligated to the transmembrane domain embedded within the nanodisc lipid bilayer [51]. This study also showed that the membrane-bound Bcl-x_L_ closely resembles the soluble form, and retains high affinity binding to a BH3 peptide (from Puma). This approach enabled a structure of the membrane-embedded transmembrane domain to be determined and showed the expected helical conformation transversing the membrane. Detailed NMR analyses enabled construction of a model in which the Bcl-x_L_ soluble domain is loosely attached to the membrane surface with the BH3 binding groove oriented towards the lipid bilayer membrane surface, as might be expected for engagement of BH3 domains from membrane-bound pro-apoptotic binding partners such as Bax and Bak. Future studies could also apply this approach to other pro-survival proteins that have been difficult to produce with their transmembrane regions to see if they interact with membranes similarly to Bcl-x_L_, and ultimately, to establish how pro-survival proteins engage with Bax and Bak in the context of the mitochondrial membrane to inhibit apoptosis.

### 4.5. Small Molecule Complexes

The development of small organic compounds with the capacity to interact with pro-survival Bcl-2 proteins and inhibit their activity was a landmark in the apoptosis field, and in the targeting of protein-protein interactions using small molecules, in general. These compounds are referred to as "BH3-mimetics" due to their capacity to mimic the action of native BH3 domain ligands. Although many compounds have been reported to be able to antagonise Bcl-2 proteins, the first ‘*bone fide*’ BH3-mimetic was ABT-737 developed by Abbott Laboratories (now AbbVie) [6]. Structural studies involving Bcl-x_L_ were instrumental in the discovery of ABT-737, as well as multiple other subsequent BH3-mimetics.

ABT-737 was discovered using “SAR by NMR”, a high-throughput NMR-based technique to screen for compounds that bound to Bcl-x_L_ [6]. Two fragments were initially identified that bound to pockets normally occupied by the h2 and h4 residues from BH3 domain peptides. These fragments were eventually connected and optimised through structure-guided design approaches. The best compound, ABT-737, has low nanomolar affinity for Bcl-x_L_ as well as Bcl-2 and Bcl-w, but not Mcl-1 or Bfl-1. Accordingly, ABT-737 was shown to have activity on Bcl-x_L_-dependent tumour cell lines and in xenograft models. Eventually ABT-737 was modified to generate an orally bioavailable analogue (ABT-263 / Navitoclax) that has been used in several past and on-going clinical trials [8]. Further modifications to ABT-263, driven by structural studies, resulted in ABT-199 (Venetoclax), which is a Bcl-2-specific derivative now used in the clinic for treatment of CLL and being tested in several other cancers [7].

Although there were multiple (NMR) structures of fragments bound to Bcl-x_L_ used in the development of ABT-737 [6], the first (crystal) structure of ABT-737 itself in complex with Bcl-x_L_ was reported later [39] (Figure 4c). This showed the chloro-biphenyl moiety engaging the h2 pocket deeper than the corresponding leucine on BH3 domains, whilst the thiophenyl group engages the h4 pocket. Although the acylsulfonamide group might have been expected to form hydrogen bonds with Arg-139, this was not seen in the structure. Rather it formed hydrogen bonds with the backbone amide of Gly-138. Binding of ABT-737 results in a widening of the hydrophobic groove, with movements of α3 and α4 similar to what was observed when BH3 peptides bind.

Subsequent to ABT-737, a number of Bcl-x_L_-specific compounds have been developed, many using structure-based approaches [24,40,42,43,45,46,47,48]. For example, the first Bcl-x_L_-selective inhibitor, WEHI-539, was developed using structure-guided design based on a compound that emerged from a high-throughput chemical library screen [40]. A more potent derivative, A1155463 used a WEHI-539 fragment that engages the h2 pocket together with SAR by NMR to screen for a more effective moiety to engage the h4 pocket [47]. Structural studies have also played a key role in informing the re-scaffolding of small molecule Bcl-x_L_-antagonists [43,45].

Multiple other structures of Bcl-x_L_ in complex with other compounds have now been determined (Table 1). Essentially, all show similar features in that the compounds engage the top-end of the groove, where the h2–h4 residues of BH3 domains normally target, and all cause the groove to widen due to similar movements of the α3-α4 helices, though to different degrees. Other obvious differences such as the depth to which the h2 pocket is entered, formation of hydrogen bonds with Arg-139 and other residues, and various hydrophobic contacts along the top portion of the groove, likely contribute to the differing affinities and selectivity of the various compounds that have been generated.

## 5. Three-Dimensional Domain-Swapped (3DDS) Bcl-x_L_ Dimers

Recent structural studies have shown that “activation” of Bax and Bak involves a significant conformational change whereby the α5-α6 helix hairpin is lost, and instead these helices form a single extended helix, releasing helices α6-α8 (referred to as the "latch" domain) that reciprocally swap with α1-α5 (“core” domain) of a second Bax or Bak molecule, creating a domain-swapped dimer [90,91]. This dimer was shown to be an “off-pathway” conformation on the transition from monomer to oligomer. Interestingly, an identical domain-swapped dimer had been reported earlier for Bcl-x_L_ following exposure to alkaline pH [50]. Subsequent studies have shown that heating the protein above 50 °C [92] (Figure 4d) and exposure to detergent (*n*-octyl-β-d-maltoside) had the same effect [18]. In contrast to Bax and Bak where BH3 peptides such as Bim and Bid could induce the conformational change, binding of BH3 peptides (and BH3-mimetics) to Bcl-x_L_ inhibited dimerisation [18,50,92], though BH3 peptides could bind to the dimer after it had formed. Whilst the physiological relevance of these Bcl-x_L_ dimers is still unclear, there is evidence that dimerisation increases pore-forming capacity in liposomes [50] and could have a role in mitochondrial calcium uptake [18].

## 6. Bcl-x_L_ Interactions with p53

Bcl-x_L_ is unique amongst pro-survival proteins in its ability to interact directly with cytosolic p53. This is believed to play an important role in the regulation of p53-induced apoptosis in response to stimuli such as DNA damage following ultraviolet irradiation, and has implications for the prevention and treatment of cancer [93,94]. In these circumstances, nuclear p53 can upregulate expression of Puma that can then engage Bcl-x_L_ [93]. This in turn results in the release of p53 that is then free to directly activate Bax [93,94]. Several studies have examined the interaction between Bcl-x_L_ and p53 in detail. NMR chemical shift data [95] delineated the p53 binding site on Bcl-x_L_ as an acidic patch adjacent to the hydrophobic groove involving the C-terminus of α1, the α3-α4 loop and α5-α6 loop. This binding site was confirmed when a solution structure of the complex was determined [96] and shown to interact with a basic surface on the p53 DNA-binding domain involved in interactions with DNA [95,96] (Figure 4e). Significant changes to the BH3 domain-binding groove due to movements in α2, α3 and α4 were also observed relative to the *apo*-Bcl-x_L_ structure, though BH3-peptide binding capacity was retained. Further NMR studies showed that binding of Puma results in a major unfolding of the α2 and α3 helices [10] that is significantly more extensive than seen when other BH3 peptides bind Bcl-x_L_. This unfolding occurs by an allosteric mechanism associated with a π-stacking interaction of a unique tryptophan residue N-terminal to the h1 residue on Puma BH3 (Figure 2a) with His-113 in the α3-α4 loop of Bcl-x_L_ [10]. Although this study showed that only Puma had the capacity to dissociate p53, an earlier NMR study [95] also showed that Bad BH3 prevented p53 binding despite not possessing a tryptophan at the same position (though it does have a tryptophan in the adjacent position) (Figure 2a). Interestingly, the same patch on Bcl-x_L_ that engages p53 is also an interaction site for the unstructured loop between α1-α2 after it undergoes post-translational modifications such as phosphorylation or deamidation [16]. Hence, such modifications potentially influence apoptotic responses by disengaging p53.

## 7. Conclusions and Future Directions

Structures of Bcl-x_L_ and the complexes it forms have underpinned much of our knowledge of how the intrinsic apoptotic pathway is regulated, and driven the discovery of small molecule antagonists that have led to potent new cancer treatments. The structural biology of most of the steps involved in the execution of the intrinsic apoptotic pathway is now well understood, though there are still some important pieces of the puzzle that are missing. These include the structure of the Bax/Bak oligomer that mediates mitochondrial permeabilisation.

Relevant to this review would be structures of Bcl-x_L_ in complex with full-length pro-apoptotic proteins (as opposed to just their BH3 domains), particularly within a membrane. Structural studies with complexes of full-length BH3-only proteins are challenging due to most (except Bid) being intrinsically disordered, though such structures could inform novel mechanisms of apoptotic regulation that do not involve the canonical BH3-in-groove interaction. For example, a recent study demonstrated that residues within the hydrophobic tail of Bim engage a (currently unknown) site outside of the BH3 binding groove on Bcl-x_L_, thereby increasing the affinity of the interaction [97]. This “double-bolt” binding mechanism has some significant implications for the mechanism(s) and utility of BH3-mimetics drugs, hence, further insights into its structural basis could be useful.

Perhaps even more interesting would be structures of Bcl-x_L_ with full-length Bax and/or Bak. These would provide important insights into how the BH3 domain of the pro-apoptotic protein is exposed to enable interaction with the Bcl-x_L_ hydrophobic groove, something that is not immediately apparent from the currently available structures. This in turn would potentially also provide insights into how Bax and Bak oligomers are assembled, as they would likely follow a topologically similar BH3-in-groove arrangement [98]. In this context, a structure of a complex between tBid and Bcl-x_L_ could potentially also provide similar information, given that Bid adopts the canonical Bcl-2 protein three-dimensional topology (unlike other BH3-only proteins).

Finally, there are a number of Bcl-x_L_ binding partners that are not core members of the Bcl-2 protein family. These include proteins such as voltage-dependent anion channel 1 and ryanodine receptors, both of which influence intracellular calcium flux [99,100,101]. Although it is known that these interactions involve the Bcl-x_L_ BH4 domain (outside of the hydrophobic groove), the precise mechanism by which Bcl-x_L_ regulates these proteins is unclear, hence, structures of these complexes could also be useful to better understand the molecular details of these processes.

Although determination of many, if not all, of these structures is likely to prove challenging, recent advances in structural biology techniques particularly in (cryo-)electron microscopy, could be of significant benefit for providing a complete high-resolution view of how the intrinsic apoptotic pathway is regulated and executed.

## Figures and Tables

**Table 1:** Structures of Bcl-x_L_ in the Protein Data Bank.

PDB ID	Structure	Type	Ref.
***Apo* Bcl-x_L_ structures**			
*Wild-type*			
1LXL, 1MAZ, 1R2D, 1AF3, 1PQ0, 3IHC, 3IIH, 2LPC, 2M03	Bcl-x_L_	X-ray, NMR	[5,9,10,11,12,13,14]
*Mutants*			
1R2E	Bcl-x_L_ E92L	X-ray	[12]
1R2G	Bcl-x_L_ F97W	X-ray	[12]
1R2H	Bcl-x_L_ A142L	X-ray	[12]
1R2I	Bcl-x_L_ F146L	X-ray	[12]
3CVA	Bcl-x_L_ W137A	X-ray	[15]
6BF2	Bcl-x_L_ S62E	NMR	[16]
3IHD, 3ILC	Bcl-x_L_ Y101A	X-ray	[13]
3IHE, 3IIG	Bcl-x_L_ F105A	X-ray	[13]
3IHF, 3ILB	Bcl-x_L_ R139A	X-ray	[13]
**BH3 domain-containing proteins in complex with Bcl-x_L_**			
*Canonical BH3 domains*			
1PQ1, 3FDL, 4QVF	Bim BH3:Bcl-x_L_	X-ray	[11,17,18]
1G5J, 2BZW	Bad BH3:Bcl-x_L_	X-ray, NMR	[19,20]
4QVE	Bid BH3:Bcl-x_L_	X-ray	[18]
2M04	Puma BH3:Bcl-x_L_	NMR	[10]
1BXL, 5FMK	Bak BH3:Bcl-x_L_	X-ray, NMR	[21,22]
3PL7	Bax BH3:Bcl-x_L_	X-ray	[23]
*Canonical BH3 domains (mutant)*			
3IO8	Bim L12F BH3:Bcl-x_L_	X-ray	[24]
4YJ4	Bim I55R/G158pS BH3:Bcl-x_L_	X-ray	[25]
5FMJ	Bak Q75L:Bcl-x_L_	X-ray	[21]
*Non-canonical BH3 domains*			
2P1L, 2PON	Beclin1 BH3:Bcl-x_L_	X-ray, NMR	[26,27]
4CIN	Bcl-x_L_ BH3:Bcl-x_L_	X-ray	[28]
4Z9V	TCTP BH3:Bcl-x_L_	X-ray	[29]
3R85	SOUL BH3:Bcl-x_L_	X-ray	[30]
6IJQ	p73-TAD:Bcl-x_L_	NMR	[31]
*Non-canonical BH3 domains (mutant)*			
6DCN, 6DCO	Beclin 1 pT108 or T108D BH3:Bcl-x_L_	X-ray	[32]
**Unnatural peptides in complex with Bcl-x_L_**			
4A1U, 4A1W	Bim-based α/β foldamers:Bcl-x_L_	X-ray	[33]
2YJ1, 4BPK	Puma-based α/β foldamers:Bcl-x_L_	X-ray	[34,35]
3FDM	Bak-based α/β foldamer:Bcl-x_L_	X-ray	[17]
2YQ6, 2YQ7, 5C3G	Bim-based hydrocarbon stapled:Bcl-x_L_	X-ray	[36,37]
2LP8	Photoswitchable Bak BH3:Bcl-x_L_	NMR	[14]
5VX3	Bim-h3Pc-RT BH3:Bcl-x_L_	X-ray	[38]
**Small molecules in complex with Bcl-x_L_**			
2YXJ	ABT-737:Bcl-x_L_	X-ray	[39]
4QNQ	ABT-263:Bcl-x_L_	X-ray	PDB only
3ZLR	WEHI-539:Bcl-x_L_	X-ray	[40]
3INQ	W1191542:Bcl-x_L_	X-ray	[24]
3ZK6, 3ZLN, 3ZLO	Benzothiazole-hydrazone ligands:Bcl-x_L_	X-ray	[40]
1YSG	SAR by NMR ligand:Bcl-x_L_	NMR	[6]
1YSI, 1YSN, 2O1Y, 2O2M, 2O2N	Acyl-sulfonamide ligands:Bcl-x_L_	NMR	[6,41]
4C52, 4C5D	Benzoylurea ligands:Bcl-x_L_	X-ray	[42]
3QKD	Quinazoline sulphoamide ligand:Bcl-x_L_	X-ray	[43]
4EHR	Pyrazole-based ligand:Bcl-x_L_	X-ray	[44]
3WIZ, 4TUH, 4QVX, 3SPF, 3SP7	Small molecule inhibitors:Bcl-x_L_	X-ray	[45,46,47,48]
**3D domain swap dimers of Bcl-x_L_ and others**			
4HNJ	Puma BH3:Bcl-x_L_ dimer	X-ray	[10]
4PPI	Detergent-induced Bcl-x_L_ dimer	X-ray	[49]
2B48	pH-induced Bcl-x_L_ dimer	X-ray	[50]
6F46	Bcl-x_L_ transmembrane domain	X-ray	[51]

## Abbreviations

Bcl	B cell lymphoma
BH	Bcl-2 homology
NMR	Nuclear magnetic resonance
PDB	Protein data bank
SAHB	Stabilised α-helix of Bcl-2 domains
SAR	Structure-activity relationships
